# Restored vision in a young dog following corticosteroid treatment of presumptive hypophysitis

**DOI:** 10.1186/s12917-017-0983-x

**Published:** 2017-02-28

**Authors:** Nina Marie Rzechorzek, Tiziana Liuti, Catherine Stalin, Katia Marioni-Henry

**Affiliations:** 1 Centre for Clinical Brain Sciences, University of Edinburgh, Edinburgh, EH16 4SB UK; 2 Royal (Dick) School of Veterinary Studies and Roslin Institute, University of Edinburgh, Easter Bush Campus, Roslin, Midlothian EH25 9RG UK; 3 The Neurology Service, Small Animal Hospital, School of Veterinary Medicine, University of Glasgow, Garscube Campus, Bearsden Road, Glasgow, G61 1QH UK

**Keywords:** Hypophysitis, Central blindness, Insulin-like growth factor-1, Magnetic resonance imaging, Pituitary tumour, Standard Poodle

## Abstract

**Background:**

Hypophysitis is an umbrella term for a group of disorders involving inflammation of the pituitary gland. A rare occurrence in humans, hypophysitis can produce a range of clinical signs including (but not limited to) visual deficits and diabetes insipidus. Only five cases of canine hypophysitis exist in the literature, all presenting in mature dogs with no visual deficits and a grave outcome. This case report describes the clinical and advanced imaging features of blindness-inducing presumptive hypophysitis in a dog, which rapidly resolved with medical management.

**Case presentation:**

A 1-year-and-seven-month-old neutered male Standard Poodle presented with subacute blindness, ataxia, and polyuria/polydipsia (PUPD). Magnetic resonance imaging (MRI) detected a contrast-enhancing pituitary mass with perilesional oedema compromising the optic chiasm. Suspecting neoplasia, anti-inflammatory corticosteroid was commenced prior to radiation therapy planning. Complete resolution of neurological and visual deficits occurred within 12 days of starting steroid treatment. Repeated advanced imaging indicated macroscopic resolution of the lesion. An extended thyroid panel with insulin-like growth factor-1 analysis supported a diagnosis of hypophysitis. Resolution of PUPD was achieved with tapering courses of prednisolone and desmopressin; the dog has since been clinically normal for 14 months and treatment-free for 11 months.

**Conclusions:**

To the authors’ knowledge, this is the first instance in which a canine pituitary mass has demonstrated long-term resolution with palliative medical treatment alone, alongside reversal of associated blindness and presumptive diabetes insipidus. We suspect this lesion to be a form of hypophysitis, which should be included among differential diagnoses for pituitary masses, and for subacute blindness in dogs. Where possible, we advocate biopsy-confirmation of hypophysitis prior to timely intervention with anti-inflammatory treatment.

## Background

Pituitary masses can represent neoplastic, cystic, infectious and/or inflammatory pathology. In dogs, the most common aetiology is neoplastic; macroadenomas, invasive adenomas and adenocarcinomas tend to present in mature animals, whilst the less common suprasellar germ cell tumour presents in younger dogs [1]. Cystic and inflammatory masses such as Rathke’s cleft cysts and immune-mediated lymphocytic hypophysitis occur far less frequently [1]. These lesions are however important to identify, given their potential to have a radically different prognosis relative to neoplasia if managed appropriately [2, 3]. Differentiating each of the above aetiologies using advanced imaging is problematic, since they exhibit variable and overlapping radiological features. For example with MRI, all of these masses can appear T2-weighted (T2w) hyperintense to normal grey matter, with variable T1-weighted (T1w) intensity, contrast enhancement, and varying degrees of perilesional oedema; neoplastic lesions can also have secondary cystic or haemorrhagic foci [1]. In humans, typical MRI features of hypophysitis include symmetrical pituitary enlargement, loss of normal neurohypophyseal T1w hyperintensity, and homogeneous contrast enhancement [1], however complete radiological descriptions of canine hypophysitis are lacking [1, 4, 5].

Clinical signs associated with pituitary neoplasms depend on their size and secretory properties; an important tumour in the dog is the corticotroph adenoma resulting in hypercortisolism [2]. Non-functional pituitary masses become clinically relevant when their mass effect induces pituitary dysfunction (leading to a range of endocrinopathies such as hypothyroidism and central diabetes insipidus) and/or neurological signs including changes in mentation, behaviour, or appetite, abnormal gait, seizures, visual loss and other cranial nerve deficits [2, 6]. Human hypophysitis is subclassified according to the structures affected (adenohypophysitis, infundibulo-neurohypophysitis or pan-hypophysitis), and can be primary (autoimmune), or secondary to systemic disease, local lesions or immunomodulatory drugs [7]. Primary hypophysitis is further categorized histopathologically as lymphocytic, granulomatous or xanthomatous – however the pathogenesis of each remains ill-defined [3]. Lymphocytic and granulomatous hypophysitis typically disrupt adrenal, gonadal and thyroidal axes, and produce diabetes insipidus and visual deficits [3]. Only five cases of canine hypophysitis have been reported (Table 1); none of these presented with visual deficits and all resulted in a grave outcome [4, 5, 8–11]. The aim of this report is to describe the successful treatment of presumptive hypophysitis in a dog, resolving all associated clinical signs including blindness and suspected diabetes insipidus. Further we provide advanced imaging details of the mass lesion before treatment and after apparent macroscopic resolution. Finally we compare and contrast our findings to previously reported cases in this species as well as humans, and discuss factors that may contribute to the development of hypophysitis.

**Table 1:** Reported cases of hypophysitis in the dog

Signalment	Presentation	Treatment	Outcome	Diagnosis	Reference
9 y MN Samoyed	3 y dermatitis and blepharitis	Medical therapy	Euthanasia	Lymphoplasmacytic adenohypophysitis and sebaceous adenitis	[10]
4.5 y FE Great Pyrenees	2 m progressive paresis and pelvic limb muscle atrophy	Prednisolone Levothyroxine	Euthanasia	Lymphocytic adenohypophysitis and adrenalitis with polyendocrine syndrome^a^	[8]
10 y FE Crossbreed	3 m progressive anorexia and weight loss; gastroenteritis, pyrexia	IVFT Ranitidine Fenbendazole	Sudden death	Lymphoplasmacytic adenohypophysitis with adrenal insufficiency and giardiasis	[11]
8 y ME German Longhaired Pointer	Acute onset PUPD, exercise intolerance, dull mentation and hair coat	Desmopressin Hypophysectomy Mannitol	Euthanasia	Lymphocytic hypophysitis with central diabetes insipidus	[5]
6 y MN Scottish Terrier	One week progressive lethargy, anorexia, pelvic limb ataxia	Not reported	Death after acute severe hypernatraemia	Lymphocytic panhypophysitis with extension to hypothalamus and polyendocrine syndrome^b^	[4]

^a^hypothyroidism and hypoadrenocorticism; ^b^suspected primary hypoadrenocorticism and secondary hypothyroidism; *PUPD* polyuria/polydipsia, *IVFT* intravenous fluid therapy, *MN* neutered male, *FN* neutered female, *ME* intact male*, FE* intact female. Note that the sellar xanthogranuloma with polyendocrine syndrome in a 7 year-old neutered male Standard Poodle reported by Cramer et al. [9] is not included in the table due to current controversy regarding the classification of this as a separate entity from hypophysitis, based on pathogenesis

## Case presentation

**Fig. 1:**
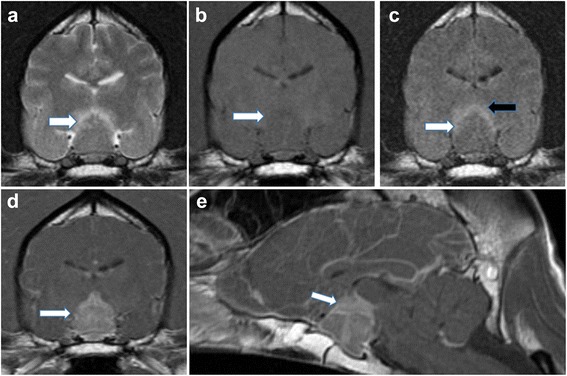
Transverse T2w, T1w, FLAIR and T1w post-contrast (**a**, **b**, **c**, **d**) and sagittal T1w post-contrast (**e**) magnetic resonance images through the pituitary fossa. Note, in all sequences, the enlarged pituitary gland (white arrow) with associated perilesional brain oedema (black arrow) in the FLAIR image (**c**). Note the avid contrast enhancement of the enlarged pituitary gland on T1w transverse and sagittal post-contrast images (**d**, **e**). Images were acquired with a 1.5 T Magneton Essenza MRI scanner

A 1-year-and-seven-month-old neutered male Standard Poodle presented to a primary veterinarian with PUPD, shivering and mild pyrexia (39.3 °C). Ongoing medication included oclacitinib maleate^1^ (0.45 mg/kg orally every 12 h) for atopic dermatitis. A urine dipstick test was normal, but after two days without improvement, amoxicillin-clavulanate^2^ (15.8 mg/kg orally every 12 h for 14 days) and meloxicam^3^ (100 μg/kg orally every 24 h for 5 days) were prescribed. 6 days later, the dog re-presented with 4-limb ataxia and blindness. He was subdued and remained polydipsic, with a reduced appetite. Haematology and biochemistry did not yield any clinically significant data, and an adrenocorticotrophic hormone (ACTH) stimulation test was within normal limits. At referral, pale mucous membranes and bradycardia (48 bpm) were noted. Neurological examination revealed visual loss with absent menace responses bilaterally, marked bilateral mydriasis and partially responsive pupillary light reflexes (PLRs). Ophthalmological examination and electroretinography confirmed normal ocular anatomy and normal retinal function respectively. Magnetic resonance imaging (Fig. 1) revealed a T1w isointense, homogeneously contrast-enhancing, symmetrical, dumbbell-shaped pituitary mass of 15 mm height (pituitary height/brain area [P/B] ratio of 0.85 [12]), with moderately increased perilesional signal on T2w images that did not suppress on fluid-attenuated inversion recovery (FLAIR). The lesion was also isointense on T2*, and together with the oedema exerted a mass effect on the optic chiasm. Cisternal cerebrospinal fluid (CSF) analysis yielded a lymphocytic pleocytosis (total nucleated cell count 34 per μl with 85% lymphocytes; protein concentration 49 mg/dL) which, alongside MRI findings, suggested a neoplastic, immune-mediated or infectious process. The most likely differential in a dog was pituitary neoplasia.

The owner elected for medical management and radiation therapy. Three days after commencing prednisolone^4^ (0.91 mg/kg orally every 24 h), PLRs were intact, and the visual function and gait were improving. Polydipsia however persisted, and urinalysis showed an inactive sediment with urine specific gravity (USG) of 1.010. Partial central diabetes insipidus and/or the influence of prednisolone treatment were considered as potential contributing factors. After a further 9 days, the neurological signs had abated; the dog was completely visual but expressed marked PUPD. Vasopressin analogue, desmopressin^5^ was commenced at 10 μg/kg (4 drops in each eye every 12 h). The dog was transferred a week later to a second referral hospital for radiation therapy planning. The PUPD had improved, but there was generalized muscle atrophy and weight loss (of 1.8 kg), with a body condition score of 3/9. Neurologically the dog was normal. Repeated haematology and biochemistry revealed increased cholesterol (321 mg/dL; reference range 147–271 mg/dL) and a few reactive lymphocytes. Urinalysis confirmed isosthenuria (USG 1.009). Computed tomography of the head and thorax detected no evidence of metastatic disease and the pituitary gland was normal in size, suggesting resolution of an inflammatory lesion.

**Fig. 2:**
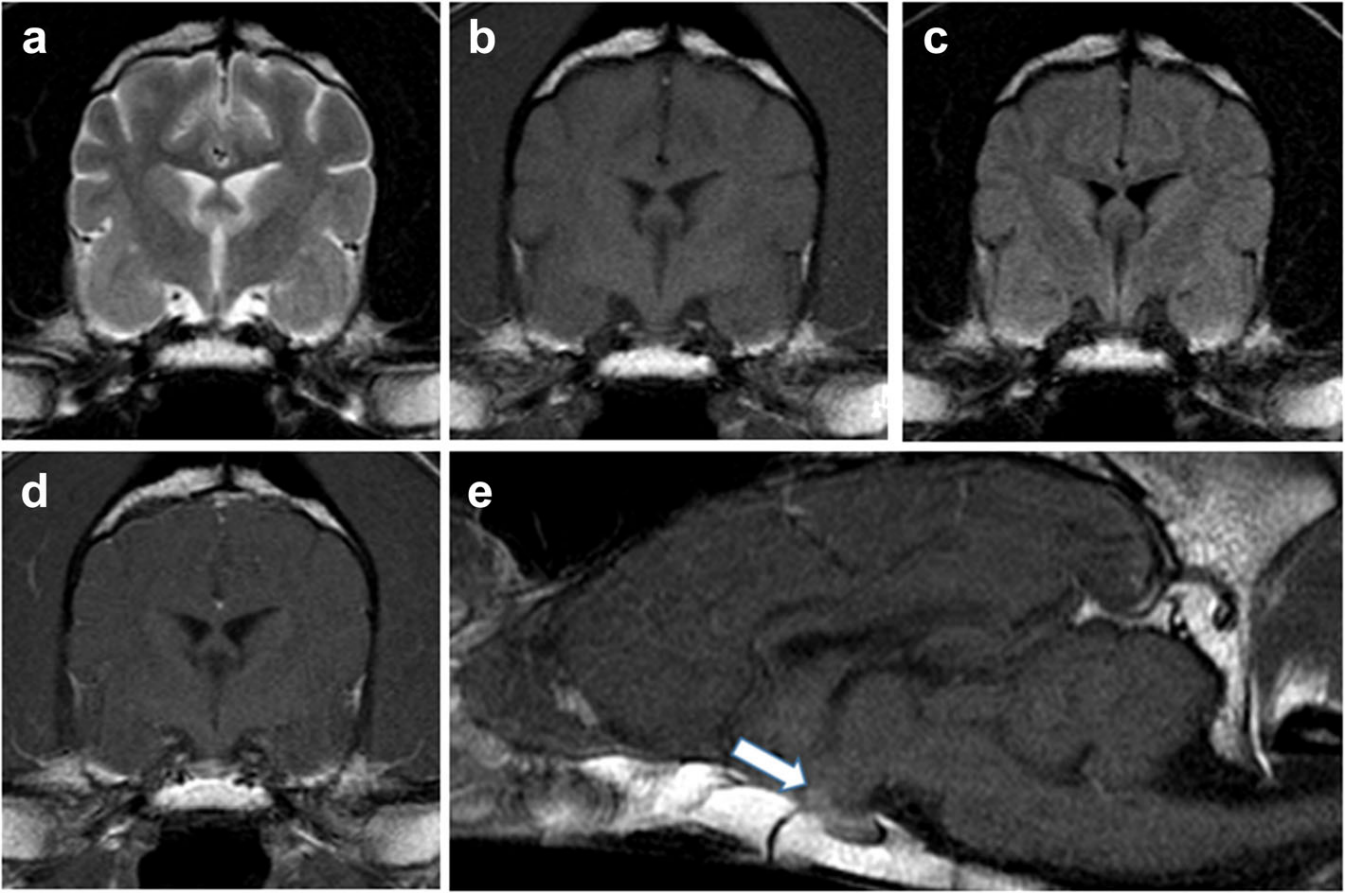
Transverse T2w, T1w, FLAIR and T1w post-contrast (**a**, **b**, **c**, **d**) and sagittal T1w post-contrast (**e**) magnetic resonance images through the pituitary fossa. Note, in all sequences, the normal appearance of the pituitary gland in the pituitary fossa; pituitary height was 4 mm. Note the contrast enhancement of the normal pituitary gland (white arrow) on T1w sagittal post contrast image (**e**). Images were acquired with a 1.5 T Philips Burgess Diagnostic MRI scanner

Two days later the owner reported further improvement with prednisolone and desmopressin; oclacitinib maleate was being given at maintenance frequency (once daily). Repeated MRI (Fig. 2) indicated complete macroscopic resolution of the mass (pituitary height had reduced from 15 mm to 4 mm with a P/B ratio of 0.23) and was considered to be within normal limits [1, 12]), with no apparent residual oedema or compression of surrounding tissues. We were not aware of any literature reporting such a dramatic response of a canine pituitary neoplasm to palliative medical treatment alone [2]. On this basis, the differentials included primary hypophysitis, iatrogenically-induced hypophysitis [7], hypophysitis secondary to a ruptured cyst of Rathke’s pouch [1, 13] or an infection [1, 10], and sellar xanthogranuloma [9]. Serology for *Toxoplasma* and *Neospora* was negative, whilst endocrine serology (Table 2) was consistent with hyposomatotropism, which can occur secondarily to hypophysitis [5]. Given the possibility of an immune-mediated aetiology, oclacitinib maleate was curtailed and prednisolone was tapered over 10 weeks. Two weeks later the dog had gained weight; after a further 4 weeks his urine was hypersthenuric and the PUPD had resolved. Desmopressin was tapered over 5 weeks. During a 14-month follow-up, there has been no recrudescence of the clinical signs and the dog has been without treatment for 11 months.

**Table 2:** Endocrinology panel^a^

Analyte	Value	Unit	Reference range
Thyroxine (T4) by RIA^b^	16.6	nmol/l	13–52
Free T4 equilibrium dialysis (RIA^b^)	7.1	pmol/l	7–40
TSH by IRMA^c^	0.23	ng/ml	<0.41
Thyroid antiglobulin antibody	52	%	<200
IGF-1	47	ng/ml	>200 adults of large breeds
			>500 puppies
			<50 dwarfism
			>1000 acromegaly

^a^(NationWide Specialist Laboratories, Cambridge; Canine Platinum Thyroid Profile and IGF-1); ^b^radioimmunoassay; ^c^immunoradiometric assay. Note low IGF-1 and borderline low free T4, with normal total T4, TSH and thyroid antiglobulin antibody. *TSH* thyroid stimulating hormone, *IGF-1* insulin-like growth factor-1

## Discussion

The rapid emergence of subdued behaviour, reduced appetite, and blindness described above resemble common symptoms of hypophysitis in humans including headache and visual disturbances [3]. These signs are also consistent with a histpathologically-confirmed case of lymphocytic panhypophysitis in a Scottish Terrier [4]; visual dysfunction was not reported in that dog, which could be due to a more dorsal (rather than rostral) extension of the lesion relative to our case [4]. By contrast, canine pituitary neoplasms tend to present more insidiously, and visual dysfunction would likely only be seen as a late-emerging sign in the few cases that had gone untreated, or non-functional tumours that had remained clinically silent for a prolonged period [2]. Occasionally, pituitary apoplexy can present secondary to acute haemorrhage within a neoplastic lesion [14], but the lack of T2* signal void in our case makes haemorrhage highly unlikely. Magnetic resonance is the preferred imaging modality for hypophysitis in humans, but has only been described for two dogs, both of which had inflammatory extension to other parts of the brain [1, 4]. The isointensity of the canine mass on T1w images, together with marked contrast enhancement and lack of cystic changes is consistent with a lymphocytic or granulomatous subtype [3]. The shape, hyperintensity on T2w sequences, and lack of T2* signal void are additional features of this lesion which match those described by Oliveira et al. [4]. By contrast both to our report and the human literature, the authors refer to a hypointensity (rather than a loss of normal pituitary hyperintensity) on pre-contrast T1w images [1, 4]. As expected due to its rich vascular supply, the normal post-treatment pituitary gland in our Standard Poodle remained markedly contrast-enhancing relative to brain tissue, with a focal T1 hyperintensity and isointensity to cortical grey matter on T2w sequences (Fig. 2) [1]. Alongside the CSF results, our case is therefore most consistent with a lymphocytic pan-hypophysitis [3, 5, 9] – although confirmation of hypophysitis and its subclassification would require histopathology. In humans, a diagnosis of primary hypophysitis is often based on relevant symptoms together with MRI findings because surgery is not needed for resolution [7]. Since the dog’s clinical signs and imaging abnormalities responded so dramatically to medical management, an invasive biopsy could not be justified. Likewise, transsphenoidal hypophysectomy can be complicated for these lesions [5] and the large P/B ratio of the mass would have increased the risk of post-operative mortality for hypophysectomy [15]. Although bacterial and canine distemper virus infections were not completely excluded, the authors consider these to be extremely unlikely given the nature and course of clinical signs in this dog.

Adissu et al. [8] likened their hypophysitis case to ‘Schmidt’s syndrome’ – a human condition comprising 2 out of 3 of the major endocrine disorders (hypoadrenocorticism, hypothyroidism and type I diabetes mellitus), and recently described in a Dobermann Pinscher [16]. Polyendocrinopathy was not confirmed in our Standard Poodle, but extended endocrine serology was performed after corticosteroid treatment had commenced, which may have impacted upon endocrine axes, and thus serum levels of the parameters tested. Despite this, the free T4 was borderline low (Table 2) and overall, the thyroid profile was almost identical to that of a Great Pyrenees with lymphocytic adenohypophysitis and confirmed polyendocrinopathy [8]. If treatment had been delayed further in our case, it is possible that hypothyroidism may have emerged as part of the clinical picture, or at least serologically. Our IGF-1 and free T4 results are also consistent with another histopathologically-confirmed example of canine lymphocytic hypophysitis [5]. Although prednisolone may have altered the thyroid profile, it should not affect serum IGF-1 in the dog [17]. Clinically therefore, our case is most similar to the Pointer with lymphocytic hypophysitis, in which hypothyroidism and hyposomatotropism occurred secondarily to hypopituitarism, and diabetes insipidus responded to desmopressin [5]. The delayed resolution of PUPD in our Standard Poodle might have resulted from pituitary stalk damage or prednisolone treatment. Absence of visual deficits in the Pointer could be attributed to a slightly smaller P/B ratio of the pituitary mass (0.75 measured on CT images) [5], which in our case was large enough to compress the optic chiasm and potentially also impinge on the optic tracts. The contrasting outcome between these two cases may reflect a combination of prompt administration of corticosteroid (rather than surgical debulking [5]), and the young age of our dog. Irrespectively, it is important to emphasise that without histopathological confirmation, our diagnosis of hypophysitis remains presumptive.

Treatment for human hypophysitis has included debulking, glucocorticoids, azathioprine, methotrexate and radiation therapy [3]. Corticosteroid is recommended initially for lymphocytic or granulomatous forms where vision is not at risk, but steroid can fail to produce long-term resolution [3]. Pre- or post-surgical glucocorticoid reduced pituitary size in 75% of patients with the lymphocytic form of hypophysitis, but was less effective in other subtypes [3]. Neoplasia is not excluded by mass size reduction and resolution of visual deficits, which can be achieved medically in patients with aggressive pituitary adenomas [18]. Functional pituitary adenoma was however ruled out in this dog by the combined results of endocrinological testing [5], the extent of radiological change, and the clinical improvement following medical management alone [2]. Radiation therapy is typically recommended for canine pituitary neoplasms, but there is little evidence that it can reverse any visual dysfunction associated with these [2, 6]. For dogs that receive no therapy or only symptomatic treatment (which may include corticosteroid), median survival time for pituitary neoplasia ranges from 6 days to 359 days [2, 19], and our patient is clinically normal beyond 400 days. One potential caveat is lymphoma, which can affect the pituitary [20] and can respond dramatically to corticosteroid (started at immunosuppressive doses) [21]. However, radiological features of primary pituitary lymphoma are quite distinct from our observations [22, 23] and canine lymphoma would generally be expected to progress within 11 months in the absence of specific treatment [2]. There is currently insufficient data to comment on the anticipated response and survival time when canine central nervous system lymphoma is treated with prednisolone as a single agent [19].

Reported cases of canine hypophysitis have ubiquitously resulted in death or euthanasia [4, 5, 8–11]. This does not necessarily imply a poor prognosis; in the past, histological confirmation could be achieved only at necropsy [4, 8, 10, 11]. Although surgical biopsies of pituitary masses have been introduced in veterinary medicine [24], they are rarely performed due to financial and technical limitations – the one reported attempt at hypophysectomy specifically for canine hypophysitis resulted in peri-operative euthanasia [5]. In most of the cases listed in Table 1, anti-inflammatory treatment was not attempted based on the suspicion of a neoplastic process. Prednisolone was administered to one dog, but not until 2 months after the onset of clinical signs [8]. We cannot presume that every hypophysitis case would be steroid-responsive; ideally surgical biopsy should be recommended prior to considering treatment options for pituitary masses. However where biopsy is not possible (and/or consent is withheld), it may be prudent to institute corticosteroid as soon as hypophysitis is suspected, especially in otherwise healthy dogs.

Relative to previous cases, our Poodle was remarkably young; we consider therefore that some trigger may have predisposed him to this condition prematurely. Notably, oclacitinib maleate treatment commenced prior to the onset of signs, and twice-daily dosing continued beyond the recommended loading period [25]. We have no evidence that oclacitinib maleate acts as an inciting or permissive factor for this disorder. We would however caution against the use of janus kinase inhibitors where potentially life-threatening immune-mediated conditions are suspected, and where these have developed alongside treatment. Iatrogenically-induced hypophysitis is recognised in human patients treated with ipilimumab [26], sitting within a spectrum of endocrinological side effects of immune checkpoint inhibitors and tyrosine kinase inhibitors [27, 28]. In most instances, this can be managed with glucocorticoids and hormone replacement therapy [29]. Interestingly, polydipsia, anorexia, lethargy and increased serum cholesterol were reported side effects in early field trials of oclacitinib maleate in the dog [25]. However, features that make iatrogenically-induced hypophysitis less likely in this case include a marked (rather than a moderate) pituitary enlargement, visual loss, and suspected diabetes insipidus [7].

Genetic diversity of the Standard Poodle is unevenly distributed across the dog leukocyte antigen (DLA) class I and II regions, such that the most inbred dogs are more susceptible to complex autoimmune diseases [30]. Among these, sebaceous adenitis and hypoadrenocorticism have presented alongside hypophysitis in other breeds [8, 10]. One suggested mechanism is that an aberrant immune response targets a unique surface antigen shared by cells of the adenohypophysis and sebaceous glands [10], however this remains untested. Cramer et al. [9] reported sellar xanthogranuloma in a Standard Poodle which also had mild hypercholesterolaemia, a low free T4, and behavioural changes [9]. However, by contrast to the case reported here, the presentation included a year of premonitory PUPD and cystic cavities within the mass [9]. Overall, the combination of atopy, thyroxine dysregulation, hyposomatotropism and hypophysitis in a Standard Poodle raises suspicion of an immune-mediated syndrome linked to the DLA region [30]. This is of interest from a classification perspective, since the co-existence of other autoimmune disorders in humans tends to occur only with the lymphocytic form of hypophysitis [3].

## Conclusions

In conclusion, we describe a presumptive case of hypophysitis in a dog with associated blindness, hyposomatotropism and suspected central diabetes insipidus. All clinical signs and radiological findings resolved after treatment with corticosteroids and the dog remains clinically normal 14 months following diagnosis, and 11 months following discontinuation of all treatments. Features that distinguish this case from previously reported instances of canine hypophysitis include a relatively young age at presentation, and long-term resolution in response to early medical management. Although visual deficits are noted frequently in human hypophysitis and sometimes with other canine pituitary masses [2, 3, 6], this is the first report of presumptive hypophysitis-induced blindness in the dog. MRI features of hypophysitis have been reported sporadically in canines [1, 4] and are similar to those described here. However, these radiological features are not specific for the disorder and cannot exclude other more common canine pituitary mass lesions. For this reason, the authors cautiously favour biopsy-confirmation of hypophysitis followed by anti-inflammatory corticosteroid and/or radiation therapy if available. Where biopsy is contraindicated, or where severe clinical signs demand urgent management, glucocorticoid might be considered. Though rare, hypophysitis should be included as a differential for pituitary lesions in dogs, including those presenting with visual loss.

## Abbreviations

CSF: Cerebrospinal fluid
CT: Computed tomography
FLAIR: Fluid-attenuated inversion recovery
IGF-1: Insulin-like growth factor-1
MRI: Magnetic resonance imaging
P/B: Pituitary height/brain area
PLR: Pupillary light reflex
PUPD: Polyuria/polydipsia
T1w: T1-weighted
T2w: T2-weighted
USG: Urine specific gravity

## Footnotes

1 Apoquel, Zoetis UK Limited, Surrey, UK.

2 Clavaseptin, Vetoquinol, Buckingham, UK.

3 Metacam 1.5 mg/ml oral suspension for dogs, Boehringer Ingelheim Limited, Berkshire, UK.

4 Prednidale, Dechra Veterinary Products.

5 DDAVP 100 μg/ml solution.
